# Bactericidal activities and biochemical features of 16 antimicrobial peptides against bovine-mastitis causative pathogens

**DOI:** 10.1186/s13567-024-01402-x

**Published:** 2024-11-14

**Authors:** Hye-sun Cho, Dohun Kim, Hyoim Jeon, Prathap Somasundaram, Nagasundarapandian Soundrarajan, Chankyu Park

**Affiliations:** Department of Stem Cell and Regenerative Biotechnology, Konkuk University, Hwayang-Dong, Kwangjin-Gu, Seoul, Republic of Korea

**Keywords:** Bovine mastitis, antimicrobial peptides, Cathelicidins, bacteriocins, biochemical properties

## Abstract

Mastitis, often caused by bacterial infection, is an inflammatory condition affecting the mammary glands. The condition is particularly prevalent in dairy cattle. Current treatment of bovine mastitis heavily relies on the use of antibiotics. To identify alternative solutions to antibiotic use, we evaluated the antimicrobial activity of 14 cathelicidins reported from 10 animal species. In conjunction, we assessed two bacteriocins against the bovine-mastitis causative bacterial panel, consisting of *Escherichia coli*, *Pseudomonas aeruginosa*, *Staphylococcus aureus*, *Bacillus cereus*, *Enterococcus faecalis*, *Streptococcus agalactiae*, *Streptococcus dysgalactiae*, and *Streptococcus equi.* Among the antimicrobial peptides (AMPs), cc-CATH3, ML-CATH, and PD-CATH proved to be highly active (minimum inhibitory concentration of 2–41 μg/mL, 0.2–10.3 μM) against all bacterial strains in the panel and field isolates from milk, with elevated somatic cell counts (≥ 500,000 cells/mL). Of the AMPs tested in this study, ML-CATH presented the highest level of effectiveness in controlling mastitis-associated bacterial strains while also possessing minimal cytotoxicity and functional stability against pH change and a high salt condition. The results of in silico analyses on the biochemical features of 12 helical cathelicidins revealed that the charge of AMPs appears to be a major determinant in killing Gram-negative bacteria. Furthermore, we observed a unique motif, “N_(n≥3)_-P_(n≥1)_-N_(n≥3)_”, from the sequences of PMAP-36, cc-CATH3, ML-CATH, and PD-CATH that exhibits potent antimicrobial activity against a broad spectrum of bacteria compared to others. Our findings support the proposition that AMPs could serve as effective antimicrobial alternatives to conventional antibiotics in treating complex animal diseases caused by microbial infection, such as bovine mastitis.

**Supplementary Information:**

The online version contains supplementary material available at 10.1186/s13567-024-01402-x.

## Introduction

Mastitis, a highly prevalent and costly disease for the dairy industry, is an inflammatory process of the mammary gland primarily caused by microbial infection [1]. Mastitis-causing bacteria are classified as either contagious or environmental microorganisms, depending on their distribution and interaction with the teat and teat duct [2]. Contagious mastitis can be transferred from cow to cow and is caused by bacterial species such as *S. aureus*, *S. agalactiae*, *S. dysgalactiae*, and *Corynebacterium* spp. In contrast, environmental pathogens such as coliform bacteria, other streptococci, *Enterococcus* spp. and *Pseudomonas* spp., which are considered opportunistic pathogens responsible for subclinical mastitis, can be spread to other cows through a contaminated barn environment and the milking process [3–5]. Consequently, contagious mastitis is typically controlled in dairy herds by post-milking teat dipping, total dry cow therapy, culling, therapeutic treatment, and proper maintenance of the milking equipment [6]. Clinical mastitis caused by environmental pathogens is the leading issue for well-managed low-somatic cell count (SCC) herds. In addition, environmental mastitis affects all dairy herds, and its relative importance increases even when contagious mastitis is under control [7].

Several strategies have been proposed to manage mastitis in herds, such as diagnosing the condition, separating affected animals, and improving hygiene and treatment protocols. Some of these practices have been implemented to improve dairy cattle health, milk quality, food safety, well-being, and farm productivity. However, the treatment for bovine mastitis relies heavily on the use of antibiotics [3, 8]. While dry cow antibiotic therapy has helped reduce the incidence of mastitis, the emergence of antibiotic-resistant pathogens has raised serious concerns [3]. Considering the complexity of the disease, reflected by the numerous causative pathogens, the variety and magnitude of the physiological responses, and the variation in the efficacy of control measures, alternative approaches are much needed [3]. Up to 200 different microbes have been isolated from mastitis cases, including primarily bacteria, fungi, and even monocellular algae. Nonetheless, staphylococci, streptococci, and coliforms appear to be most commonly associated with intramammary infections [4, 9, 10].

In this study, we propose that endogenous antimicrobial peptides (AMPs) can provide an alternative approach to combat the threat from infectious diseases caused by bacterial pathogens, including multidrug-resistant (MDR) bacteria. AMPs are evolutionarily ancient and pervasive components of the innate immune system in multicellular organisms such as animals, plants, and insects [11]. To date, several AMP families have been identified, either derived from a common ancestor (belonging to a genetic family) or having independently evolved from similar properties in primary or secondary structures [12, 13]. These peptides exhibit direct antibacterial effects against various microbes due to the cationic and amphipathic structures capable of disrupting the microbial membrane [14].

AMPs can be divided into many subtypes according to different criteria, such as their origin, size, structure, amino acid sequence, biological action, acting mechanism, and conserved motif [15, 16]. Despite this division, most AMPs possess positively charged residues, with a minor subgroup containing anionic amino acids [17]. Their secondary structure further categorises them into four classes: alpha-helical, beta-sheet, extended, and looped peptides [18]. Several AMPs of interest have been engineered to enhance antimicrobial activity and reduce their cytotoxic effects based on their structural and biochemical properties [19–21]. However, our understanding of these molecules’ biological functions and mechanisms is still limited.

For this study, based on available information from previous research, we selected two bacteriocins and 16 AMPs comprising 14 cationic cathelicidins from 10 animal species. We evaluated their antimicrobial activities against the bacterial panel associated with bovine mastitis, including field isolates. We also tested these molecules’ cytotoxicity and functional stability in different physiological conditions. As a result, we presented a suitable AMP candidate for controlling a broad spectrum of mastitis-causing bacterial species in cattle. The information accumulated on the functional characteristics of the AMPs compared to the structural and biochemical characteristics and peptide sequences of the individual AMPs in this study contributes to developing a new class of therapeutic agents capable of controlling diverse bacterial diseases in animals.

## Materials and methods

### Preparation of antimicrobial peptides

Using a commercial service (GenScript, Piscataway Township, NJ, United States), AMPs, including ΔModoCath1, ΔModoCath5, ΔModoCath6, PMAP-36, PR-35, ΔPb-CATH4, ΔHg-CATH, BMAP-28, EA-CATH1, cc-CATH3, HA-CATH, ML-CATH, and PD-CATH were synthesised by solid-phase peptide synthesis and purified by high-performance liquid chromatography (HPLC). Bacteriocins, nisin, and lysostaphin were purchased commercially (Sigma Aldrich, St. Louis, MO, USA; chemicals without source information in this study were purchased from Sigma Aldrich).

Recombinant PG1 was produced by employing previously reported methods of green fluorescent protein (GFP)-scaffold system [22, 23]. In brief, the DL4GFP-PG1 construct-transformed BL21 (DE3) cells were grown in 1 L of Luria–Bertani (LB) medium (BD Bioscience, Franklin Lakes, NJ, USA) at 37 °C and 220 rpm. To induce the expression of GFP-PG1 fusion protein, 0.1 mM isopropyl *β*-d-1-thiogalactopyranoside at 0.8–1.0 OD_600_ was used. After 5 h of induction, cells were harvested by centrifugation at 9900 × *g* for 10 min at 4 °C and then disrupted by sonication (Sonopuls HD 2070; Bandelin, Berlin, Germany) in the lysis buffer (20 mM sodium phosphate buffer at pH 7.4 containing 150 mM sodium chloride, 0.1 mM phenylmethylsulfonyl fluoride, and 1 mM dithiothreitol).

The insoluble fraction was separated by centrifugation at 20 000 × *g* for 20 min at 4 °C. To remove cellular debris, nucleic acids, and cytosolic proteins, DNase (0.01 mg/mL), lysozyme (0.1 mg/mL), and 0.5% Triton-X 100 were added to the insoluble fractions and incubated at room temperature (24 °C) for 20 min. The pellets were washed thrice in the lysis buffer without adding DNase and lysozyme. The insoluble fraction was dissolved in urea buffer (20 mM sodium phosphate buffer at pH 7.4 containing 8 M urea, 500 mM sodium chloride, and 30 mM imidazole). The target proteins were purified using affinity chromatography with the HisTrap HP column (GE Healthcare, Chicago, IL, USA). After undergoing dialysis and lyophilisation, the insoluble precipitates were dissolved in 70% formic acid and subjected to the addition of cyanogen bromide (CNBr). The mixture was then incubated for 24 h to cleave the N- and C-terminal GFPs flanking PG1.

After removing CNBr by lyophilisation, the target peptides were purified using a preparative reverse-phase HPLC (RP-HPLC) column (DeltaPak C18 Prep column 19, 300 mm; Waters, Tokyo, Japan) in a linear gradient of acetonitrile (5–90%)/0.1% trifluoroacetic acid for 60 min at a flow rate of 12 mL/min. The target peptides were collected at optical densities of 214 nm and 280 nm. The target proteins collected before CNBr cleavage and after RP-HPLC were visualised on both 12% sodium dodecyl sulfate–polyacrylamide gel electrophoresis (SDS-PAGE) and 16% Tris-Tricine PAGE, respectively. The collected RP-HPLC fractions were lyophilised and suspended in 20 mM sodium phosphate buffer at pH 7.4 containing 8 M urea, 5 mM reduced glutathione, and 0.5 mM oxidised glutathione to install disulfide bonds in PG1 peptides. After dialysis against deionised water, the mixture was lyophilised and quantified using the Bradford assay (Bio-Rad, Hercules, California, USA). The produced peptide was aliquoted and stored at -20 °C until use.

### Standard bacterial strains and culture

The standard bacterial strains used for antimicrobial activity assays include *Staphylococcus aureus* ATCC (American Type Culture Collection, Manassas, VA, USA) 6538, *Bacillus cereus* ATCC 10876, *Enterococcus faecalis* ATCC 29212, *Streptococcus agalactiae* ATCC 27956, *Streptococcus dysgalactiae* ATCC 27957, *Streptococcus equi* subsp. *zooepidemicus* ATCC 43079, *Escherichia coli* ATCC 25922, and *Pseudomonas aeruginosa* ATCC 27853. Most bacterial strains were cultured in LB medium except for *E. faecalis* and streptococci, which were cultivated in brain heart infusion (BHI) broth (BD Bioscience).

### Isolation of bacteria from milk

From a commercial dairy herd in Ansung, South Korea, and in line with the Korean Ministry of Food and Drug Safety (MFDS; 2018) protocol, a total of 56 milk samples with elevated SCC (≥ 500,000) were collected from 14 cows experiencing subclinical mastitis. The first streams of milk were discarded, and the subsequent samples were stored below 4 ℃. A 1 mL aliquot of the samples was subject to cultivation within 24 h after collection. The remaining aliquots were immediately frozen at − 20 ℃ for future experiments. The tenfold serial dilutions in 0.9% saline (Sigma-Aldrich) were conducted in triplicate, and bacteria were isolated and enumerated using the spread plate method. The serial dilutions were plated onto (i) Mueller–Hinton (MH) agar at 37 ℃ for 24 h; (ii) MacConkey agar (MCA) at 37 ℃ for 24 h, which selects for enterobacteria; and (iii) Tryptic soy agar (TSA) supplemented with 5% (v/v) defibrinated horse blood (MBcell, Kisanbio, Seoul, Korea) at 37 ℃ for 48 h. All plates were cultivated aerobically. Visual culture characteristics of bacterial colonies on agar plates, including haemolytic characteristics, were assessed.

### Bacterial classification using 16S ribosomal RNA sequencing

As outlined in a previous study [24], sequence analysis of the 16S small subunit ribosomal RNA gene was conducted for the taxonomic classification of field isolates. A single bacterial colony was transferred into 20 µL of sterile water in a polymerase chain reaction (PCR) tube (0.2 mL) and incubated at 100 °C for 15 min. Subsequently, 20 µL of PCR reactions comprising 2 µL of bacterial lysates, 2.0 µL of 10 × buffer (10 mM Tris–HCl, 50 mM KCl), 1.2 µL of 25 mM MgCl_2_, 2 µL of 10 mM dNTP, 0.7 µL of each primer (10 pmol/µL), and 0.2 µL of Taq DNA polymerase (1 U, SolGent, Daejeon, Korea) were conducted using a cycling profile. The profile consisted of 95 °C for 5 min, 30 cycles of 95 °C for 1 min, 50 °C for 30 s, and 72 °C for 1.5 min. The final extension was at 72 °C for 10 min.

The primer sequences for amplifying 16S rRNA were 5’-AGAGTTTGATCCTGGCTCAG-3’ and 5’-GGTTACCTTGTTACGACTT-3’ for forward and reverse, respectively [25]. The PCR products were purified using a MinElute Kit (Qiagen, Crawley, West Sussex, UK) and sequenced using AmpliTaq polymerase and BigDye Terminator (Applied Biosystems, Foster City, CA, USA) using the same primers in both directions while following the manufacturers’ instructions. The reactions were run on an ABI PRISM 3730 DNA Analyzer (Applied Biosystems). The species identity of each colony was determined using blast analysis against the National Center for Biotechnology Information (NCBI) rRNA/ITS databases [26].

### Antibacterial activity assay for antimicrobial peptides and bacteriocins

The minimum inhibitory concentration (MIC) was determined by following the manufacturer’s protocol and Clinical and Laboratory Standards Institute (CLSI) guidelines (2018) using a colourimetric method specified by the Microbial Viability Assay Kit-WST (Dojindo, Kumamoto, Japan). Four colonies of each bacterium were inoculated into 5 mL of LB medium and incubated at 37 °C for 4–6 h. The cells were then washed twice with sterile saline (0.9% NaCl) and seeded into a single well of a 96-well plate at a density of 1.5 × 10^5^ cells/well, adjusting by McFarland standard 0.5. Subsequently, 180 µL/well of fresh MH broth (BD Bioscience) was added to the plate. Ampicillin, chloramphenicol, and gentamicin sulfate were used as controls for antimicrobial activity.

Different concentrations of each peptide and reference antibiotics (up to 40 µg/mL) were serially diluted in 10 µL of MH broth and added to each well. The plate was incubated at 37 °C for 6 h, using BHI broth to culture slow-growing fastidious strains, including *E. faecalis*, *A. hydrophila*, *C. simulans*, *G. halophytocola*, and streptococci. Cation-adjusted Mueller Hinton II broth (CAMHB) was employed to cultivate *P. aeruginosa*. Afterwards, 10 µL of the colouring reagent was added, and cells were incubated at 37 °C for 2 h. UV absorbance was measured for each well at 450 nm using a microplate spectrophotometer (xMark spectrophotometer; Bio-Rad, Hercules, CA, USA). The MIC values were determined when the absorbance difference between treatments and blanks (media and colouring reagent only) decreased to < 0.05. Experiments were conducted in triplicate.

### Estimation of haemolytic and cytotoxic activity

Human keratinocytes (HaCaT) and pig kidney fibroblast cells (PK15, ATCC CCL-33) were cultured in Dulbecco’s modified Eagle’s medium (DMEM; Hyclone™, Logan, UT, USA) and supplemented with 10% foetal bovine serum (FBS; Hyclone™, Logan, UT, USA), 1% penicillin–streptomycin (Hyclone™) at 37 °C, and 5% CO_2_ up to 80% confluence. Cell substratum adhesion was disrupted using Accutase (Innovative Cell Technologies, San Diego, CA, USA). A total of 4 × 10^4^ cells were incubated for 24 h at 37 °C and 5% CO_2_ in each well of a 96-well plate containing 64 and 160 µg/mL of each peptide.

A positive control for complete cell lysis was established using Triton-X100 (1%), and untreated cells were used as the negative control. After incubation, the medium was removed from the wells and replaced with 10 µL of colouring solution (Cell Proliferation Reagent WST-1™) and 100 µL of DMEM (Hyclone™), per the manufacturer’s protocol. Each well absorbance was measured at 440 nm (peptide-treated and control) and 650 nm (background). These measurements were recorded as the optical density (OD) using a microplate reader (xMark™ spectrophotometer; Bio-Rad, CA, USA). Cell viability was calculated using the following equation:

$$Cell \,\,Viability \,(\%)=100 \times \frac{(OD \,\,peptide -OD \,\,background)}{(OD\,\, negative -OD\,\, background)}$$

All experiments were performed in triplicate.

The experimental procedures were conducted in accordance with the guidelines approved by the Institutional Animal Care and Use Committee (IACUC) of Konkuk University, Seoul, Korea, and bovine blood was collected from a cow at a local farm using a BD Vacutainer® Sodium Heparin (BD Bioscience) to analyse haemolytic activity. Plasma was removed from the whole blood by centrifugation. Erythrocytes were washed thrice with phosphate-buffered saline (PBS, pH 7.4) and resuspended in PBS at a concentration of 2% (v/v). Subsequently, 100 µL of the erythrocyte suspension was added to each well of a 96-well microtiter plate. The plate contained an equal volume of the peptides in PBS with different concentrations of 10, 20, 40, 80, and 160 μg/mL. Triton-X 100 (1%) and PBS were used as positive and negative controls, respectively. Plates were incubated at 37 °C for 1 h. The release of haemoglobin into the supernatant was monitored by measuring the change in OD at 410 nm using a microplate reader (xMark™ spectrophotometer; Bio-Rad). Results from three independent experiments were pooled. Hemolysis was calculated using the formula:

$${Hemolytic\,\, Percentage} \,(\%)=100 \times \frac{(OD\,\, peptide -OD \,\,negative)}{(OD\,\, positive -OD\,\, negative)}$$

All experiments were carried out in triplicate.

### Analysis of the functional stability of AMPs under different physiological conditions

The MICs against *E. coli* (ATCC 25922) for cc-CATH3, ML-CATH, and PD-CATH in 150 mM NaCl and different pH (pH 5, 6, and 7) that were adjusted using acetic acid (Sigma Aldrich) were determined as described above. For serum stability assay, each AMP at four different concentrations was dissolved in 50% (v/v) FBS (Hyclone™) and incubated at 37 °C for 0, 60, and 120 min, respectively. As described in a previous study [27], ΔHg-CATH was used as a control for serum sensitivity. The antimicrobial activity of cc-CATH3, ML-CATH, and PD-CATH against *E. coli* was assessed for each condition as above. All experiments were conducted in triplicate.

### In silico analysis of biochemical features

Amino acid sequences, antimicrobial activities, and structure information of publicly available cathelicidins were obtained from DBAASP (Database of Antimicrobial Activity and Structure of Peptides) [28]. The molecular weight and grand average of hydropathicity (GRAVY) scores for the primary peptide sequences of 16 selected AMPs were calculated using ProtParam [29]. The net charge value of each peptide at pH 7 was calculated using Protein Calculator v3.4 [30], assuming the p*K*a value of all residues was equal to that of the individual amino acid. Peptide 2.0 [31] was used to calculate the hydrophobic and cationic percentages. Helical wheel projections and the prediction of secondary structures and solvent accessibility (SA) for alpha-helical cathelicidins were conducted using HeliQuest [32] and I-TASSER [33], respectively. The analysis of amino acid properties constituting AMPs and visualisation of tertiary structures was performed using PyMOL [34].

### Statistical analysis

The correlation coefficient (r) between MIC and biochemical properties was calculated using the following equation [35]:

$$r\left( {x,y} \right) = \frac{{\sum {(x - \overline{x} )} (y - \overline{y} )}}{{\sqrt {\sum {(x - \overline{x} )^{2} } \sum {(y - \overline{y} )^{2} } } }}$$

Welch’s *t* test was used to test the difference in population means of MIC and biochemical properties [36].

## Results

### Identification of 13 bacterial strains from mastitis-affected milk

A total of 17 bacterial strains were isolated from culturing 56 milk samples of dairy cows with high SCC (≥ 500,000 cells/mL). Isolation involved microbiological analyses assessing growth, colony morphologies, and haemolytic characteristics on the blood agar plate (Additional file 1). Using isolated bacterial colonies, PCR generated amplicons of approximately 1500 bp in size, thus corresponding to 16S rRNA. NCBI blast analysis was conducted against rRNA/ITS databases using the sequencing results of amplicons that matched 16S rRNA sequences of known bacterial species with coverage of > 96% and identity of > 97% (Additional file 1). Multiple colonies were isolated for *Staphylococcus chromogenes* (*n* = 3), *Corynebacterium amycolatum* (*n* = 2), and *Aerococcus viridans* (*n* = 2), with 13 different bacterial strains from mastitis-affected milk identified in this study.

### Diverse physicochemical properties of 14 cathelicidins and two bacteriocins

We selected 16 AMPs comprising 12 cathelicidins from eight mammalian species, including opossums (ΔModoCath1, ΔModoCath5, and ΔModoCath6), pigs (PMAP-36, PG1, and PR-35), mole rats (ΔHg-CATH), horses (EA-CATH1), and bats (HA-CATH, ML-CATH, and PD-CATH). Additionally, we included a cathelicidin for each of the pythons (ΔPb-CATH4), quails (cc-CATH3), and two bacteriocins (nisin and lysostaphin) to identify AMPs with high therapeutic potential to bovine mastitis [27, 37–46]. The selected cathelicidins were previously reported to be highly potent against the tested bacterial strains, including *S. aureus*, *E. faecalis*, and *P. aeruginosa* at MICs < 10 μM despite the limits in available information on the activity spectrum and potency against reported bovine-mastitis causative pathogens [37, 39, 43, 44].

An in silico biochemical feature analysis of these selected 16 AMPs was conducted. For the in silico analysis of nisin, the primary sequence before post-translation modification was used as a query. Table 1 shows the amino acid sequences of each AMP and the results of the in silico biochemical feature analysis. The length and molecular weight distributions of these AMPs were 19–40 amino acids and 2.29–4.64 kDa, respectively.

**Table 1:** **The physicochemical characteristics of analysed cathelicidins in this study**

Names	Origin	Sequences	Length	Molecular weight (Da)	Net charge at pH 7	Hydrophobic residue (%)	Cationic residue (%)	GRAVY score
ΔModoCath1	*Monodelphis domestica*	VKRTKRGARRGLTKVLKKIFGSIVKKAVSKGV	32	3510.37	11.9	37.50	37.50	− 0.31
ΔModoCath5		WYQLIRTFGNLIHQKYRKLLEAYRKLRD	28	3623.27	5.1	35.71	28.57	− 0.85
ΔModoCath6		VRRSKRGIKVPSFVKKVLKDVVSESIS	27	3042.66	5.9	40.74	29.63	− 0.19
PMAP-36	*Sus scrofa*	GRFRRLRKKTRKRLKKIGKVLKWIPPIVGSIPLGCG	36	4157.22	12.9	41.67	36.11	− 0.46
PG-1^a^		MRGGRLCYCRRRFCVCVGR	19	2291.82	5.8	22.22	33.33	− 0.25
PR-35		RRRPRPPYLPRPRPPPFFPPRLPPRIPPGFPPRFP	35	4223.1	8.9	68.57	25.71	− 1.32
ΔPb-CATH4	*Python bivittatus*	TRSRWRRFIRGAGRFARRYGWRIA	24	3052.55	8.9	37.50	37.50	− 1.10
ΔHg-CATH	*Heterocephalus glaber*	SKFFRKARKKLGKGLQKIKNVLRKY	25	3035.77	10.9	32.00	44.00	− 1.08
BMAP-28	*Bos taurus*	GGLRSLGRKILRAWKKYGPIIVPIIRIG	28	3131.89	6.9	50.00	25.00	0.23
EA-CATH1	*Equus asinus*	KRRGSVTTRYQFLMIHLLRPKKLFA	25	3060.75	7.1	44.00	32.00	− 0.35
cc-CATH3	*Coturnix coturnix*	RVRRFWPLVPVAINTVAAGINLYKAIRRK	29	3379.11	6.9	58.62	24.14	0.14
HA-CATH	*Hipposideros armiger*	ILGRLRDLLRRGGRKIGQGLERIGQRIQGFFSNREPMEES	40	4640.37	3.9	32.50	22.50	− 0.82
ML-CATH	*Myotis lucifugus*	LNPLIKAGIFILKHRRPIGRGIEITGRGIKKFFSK	35	3975.87	8.1	45.71	28.57	− 0.04
PD-CATH	*Phyllostomus discolour*	ILGPALRIGGRIAGRIAGKLIGDAINRHRERNRQRRG	37	4088.79	8.2	37.84	29.73	− 0.65

^a^The peptide has an additional methionine at N-terminus as it was produced by a recombinant expression system [22].

**Figure 1:**
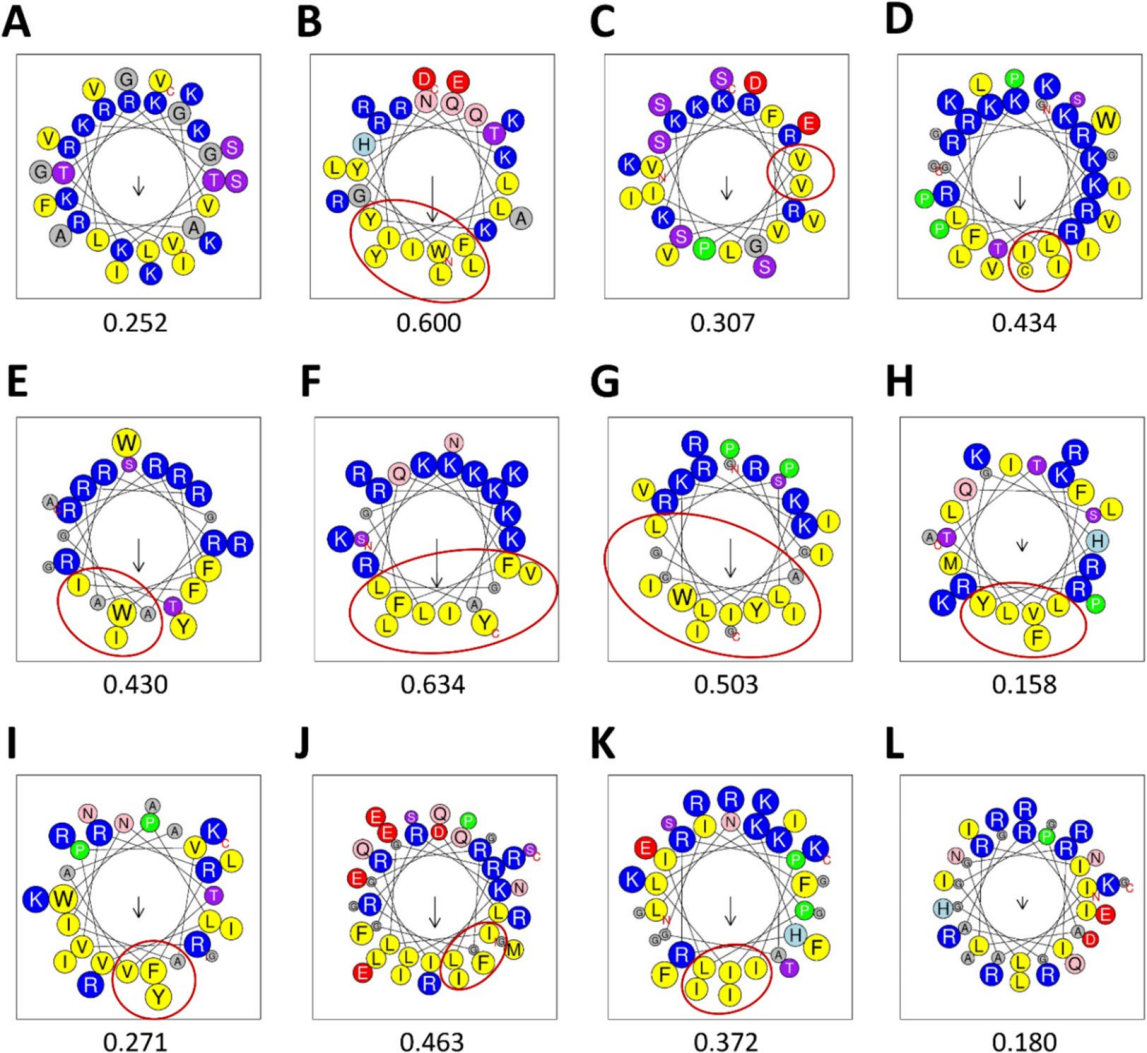
**The helical wheel modeling of 12 alpha-helical cathelicidins**. **A** ΔModoCATH1, **B** ΔModoCATH5, **C** ΔModoCATH6, **D** PMAP-36, **E** ΔPb-CATH4, **F** ΔHg-CATH, **G** BMAP-28, **H** EA-CATH1, **I** cc-CATH3, **J** HA-CATH, **K** ML-CATH and **L** PD-CATH. Helical wheel projection diagrams were created using HeliQuest with full window size and code size proportional to amino acid volume. Nonpolar residues are shown in yellow and grey, while polar (uncharged polar, negatively, and positively charged) residues are shown in purple, red, and blue, respectively. Cysteine and proline are indicated by green color. The size of the arrow (↓) represents the level of hydrophobic moment.

The hydrophobic and cationic residues ratios were 22.22–68.57% and 22.50–44.00%, respectively. The charges at pH 7 ranged from + 3.9 to + 12.9. The peptide with the highest charge at pH 7 was PMAP-36 (+ 12.9), while ΔHg-CATH possessed the highest cationic residue rate (44.0%). The grand average of hydropathy (GRAVY) index score was negative (− 1.32 to − 0.04) for most cathelicidins except BMAP-28 (0.23) and cc-CATH3 (0.14) (Table 1). The hydrophobic moment values indicating helix properties of alpha-helical cathelicidins were 0.158–0.634. However, the hydrophobic face was not predicted in ΔModoCath1 and PD-CATH, showing a small hydrophobic moment (Figure 1).

### Bactericidal activities of cc-CATH3, PD-CATH and ML-CATH against all tested bacterial species

We evaluated the antimicrobial activity of the peptides in our AMP panel against eight standard bacterial strains. The evaluation consisted of two Gram-negative strains, *E. coli* and *P. aeruginosa*, and six Gram-positive strains, *S. aureus*, *B. cereus*, *E. faecalis*, *S. agalactiae*, *S. dysgalactiae*, and *S. equi* subsp. *zooepidemicus*. In antimicrobial activity analysis against the standard bacterial strains, PR-35 and ΔModoCath6 showed activity only against *E. coli* (ATCC 25922, Table 2).

**Table 2:** **Bactericidal activities of cathelicidins and bacteriocins against 8 standard strains**

Antimicrobials	Minimal inhibitory concentration (μg/mL, μM)							
	*Escherichia coli*	*Pseudomonas aeruginosa*	*Staphylococcus aureus*	*Bacillus cereus*	*Enterococcus faecalis*	*Streptococcus agalactiae*	*Streptococcus dysgalactiae*	*Streptococcus equi* subsp.* zooepidemicus*
ΔModoCath1	4 (1.1)	10 (2.9)	11 (3.2)	> 40 (10.5)	10 (2.9)	> 40 (10.5)	> 40 (10.5)	40 (10.5)
ΔModoCath5	21 (5.8)	> 40 (11.0)	6 (1.7)	26 (7.3)	10 (2.8)	> 40 (11.0)	> 40 (11.0)	> 40 (11.0)
ΔModoCath6	25 (8.2)	> 40 (13.2)	> 40 (13.2)	> 40 (13.2)	> 40 (13.2)	> 40 (13.2)	> 40 (13.2)	> 40 (13.2)
PMAP-36	3 (0.7)	8 (1.9)	9 (2.2)	28 (6.7)	> 40 (9.6)	> 40 (9.6)	> 40 (9.6)	> 40 (9.6)
PG-1	23 (10.0)	20 (8.7)	43 (18.8)	> 40 (17.5)	> 40 (17.5)	> 40 (17.5)	32 (14.0)	> 40 (17.5)
PR-35	15 (3.6)	> 40 (9.5)	> 40 (9.5)	> 40 (9.5)	> 40 (9.5)	> 40 (9.5)	> 40 (9.5)	> 40 (9.5)
ΔPb-CATH4	5 (1.6)	11 (3.5)	4 (1.3)	> 40 (13.1)	> 40 (13.1)	> 40 (13.1)	> 40 (13.1)	20 (6.6)
ΔHg-CATH	4 (1.3)	5 (1.7)	> 40 (13.2)	> 40 (13.2)	> 40 (13.2)	> 40 (13.2)	15 (4.9)	15 (4.9)
BMAP-28	4 (1.3)	9 (2.9)	5 (1.6)	7 (2.2)	15 (4.8)	> 40 (12.6)	> 40 (12.6)	> 40 (12.6)
EA-CATH1	9 (2.9)	19 (6.1)	41 (13.4)	> 40 (13.1)	30 (9.8)	> 40 (13.1)	> 40 (13.1)	> 40 (13.1)
cc-CATH3	3 (0.9)	4 (1.28)	2 (0.6)	4 (1.28)	5 (1.5)	4 (1.28)	7 (2.0)	5 (1.5)
HA-CATH	18 (3.9)	> 40 (8.6)	26 (5.6)	25 (5.4)	> 40 (8.6)	> 40 (8.6)	> 40 (8.6)	> 40 (8.6)
ML-CATH	2 (0.5)	42 (10.6)	2 (0.5)	3 (0.8)	5 (1.3)	8 (2.0)	14 (3.5)	9 (2.3)
PD-CATH	7 (1.7)	42 (10.3)	3 (0.7)	6 (1.5)	6 (1.5)	8 (2.0)	18 (4.4)	17 (4.2)
Nisin	> 64 (19.2)	> 64 (19.2)	2 (0.60)	4 (1.2)	16 (3.6)	2 (0.60)	4 (1.2)	4 (1.2)
Lysostaphin	> 160 (29.7)	> 160 (29.7)	1 (0.2)	> 160 (29.7)	> 160 (29.7)	> 160 (29.7)	> 160 (29.7)	> 160 (29.7)
Chloramphenicol^a^	3 (9.3)	80 (247.6)	8 (24.8)	10 (31.0)	10 (31.0)	5 (15.5)	4 (12.4)	5 (15.5)
Ampicillin^a^	5 (14.3)	> 640 (1831.7)	2 (5.7)	80 (228.8)	10 (28.6)	4 (11.4)	2 (5.7)	2 (5.7)
Gentamicin^a^	1 (2.1)	1 (2.1)	1 (2.1)	1 (2.1)	90 (189.0)	75 (157.5)	15 (31.5)	45 (94.5)

^a^Used as controls.

*E. coli* ATCC 25922, *P. aeruginosa* ATCC 27853, *S. aureus* ATCC 6538 *B. cereus* ATCC 10876, *E. faecalis* ATCC 29212, *S. agalactiae* ATCC 27956, *S. dysgalactiae* ATCC 27957, *S. equi* subsp. *zooepidemicus* ATCC 43079.

Additionally, ΔModoCath5, HA-CATH, PG1, ΔPb-CATH4, EA-CATH1, ΔModoCath1, PMAP-36, BMAP-28, and ΔHg-CATH exhibited bactericidal activities against only some Gram-negative and Gram-positive strains with MICs that ranged between 3 and 43 μg/mL (0.7–18.8 μM), while lacking activity against the rest of the strains (Table 2). Out of the bacteriocins tested, lysostaphin showed specific activity against *S. aureus* with a MIC of 1 μg/mL (0.2 μM). Nisin, however, was active against all Gram-positive strains in our panel with MICs of 2–16 μg/mL (0.6–3.6 μM). In contrast, interestingly, cc-CATH3, PD-CATH, and ML-CATH showed potent and broad-spectrum bactericidal activities against all bacterial species in our test panel with MICs of 2–42 μg/mL (0.5–10.6 μM).

### Potent bactericidal activities of cc-CATH3, PD-CATH, and ML-CATH against field isolates

We also conducted an antimicrobial activity assay against field isolates from milk with high SCCs, using cc-CATH3, PD-CATH, and ML-CATH. The assay consisted of one Gram-negative (*A. hydrophila*) and 7 Gram-positive strains, including four staphylococci (*S. chromogenes*, *S. haemolyticus*, *S. xylosus*, and *S. epidermidis*), *S. uberis*, *C. simulans,* and *G. halophytocola*. As previously noted, staphylococci are a major causative bacterial genus for clinical mastitis and are commonly transmitted by contact with infected milk [3, 47]. *S. chromogenes* is also a highly prevalent species with a well-established impact on SCC in milk [48, 49]. The bactericidal potency of cc-CATH3, PD-CATH, and ML-CATH against these field isolates was similar to those among them with MICs of 0.8–5 μg/mL (0.2–1.2 μM) except *A. hydrophila* (Table 3).

**Table 3:** **Antimicrobial activity of cc-CATH3, PD-CATH, and ML-CATH against 8 field isolates from milk with high somatic cell counts**

	Strains	Minimal inhibitory concentration (μg/mL, μM)					
		cc-CATH3	ML-CATH	PD-CATH	Chloramphenicol^a^	Ampicillin^a^	Gentamicin^a^
Gram-negative bacteria	*Aeromonas hydrophila*	> 160 (47.4)	160 (40.2)	160 (39.1)	5 (15.5)	> 240 (686.4)	4 (8.4)
Gram-positive bacteria	*Staphylococcus chromogenes*	3 (0.9)	2 (0.5)	4 (1.0)	18 (55.8)	5 (14.3)	2 (4.2)
	*Staphylococcus haemolyticus*	2 (0.6)	2 (0.5)	3 (0.7)	10 (31.0)	10 (28.6)	10 (20.9)
	*Staphylococcus xylosus*	2 (0.6)	2 (0.5)	3 (0.7)	8 (24.8)	5 (14.3)	1 (2.1)
	*Staphylococcus epidermidis*	2 (0.6)	2 (0.5)	3 (0.7)	5 (15.5)	5 (14.3)	4 (8.4)
	*Corynebacterium simulans*	0.8 (0.2)	1 (0.3)	1 (0.2)	8 (24.8)	> 240 (686.4)	10 (20.9)
	*Glutamicibacter halophytocola*	2 (0.6)	2 (0.5)	1 (0.2)	3 (9.3)	15 (42.9)	3 (6.3)
	*Streptococcus uberis*	4 (1.2)	3 (0.8)	5 (1.2)	50 (155.0)	> 240 (686.4)	60 (125.6)

^a^Used as controls.

CLSI breakpoints of ampicillin against *Staphylococcus* spp., *Corynebacterium* spp., and *S. uberis* are ≥ 0.25, 0.125 and 0.6 μg/mL, respectively.

The bactericidal effect on *A. hydrophila* was observed only from PD-CATH and ML-CATH with MICs of 160 μg/mL (39.1 and 40.2 μM, respectively). This effect is much lower than the activity against other field isolates. All three AMPs demonstrated more potent antimicrobial activity against Gram-positive field isolates than the control antibiotics.

### Functional stability of cc-CATH3, PD-CATH, and ML-CATH against changes in pH and salt concentrations

**Figure 2:**
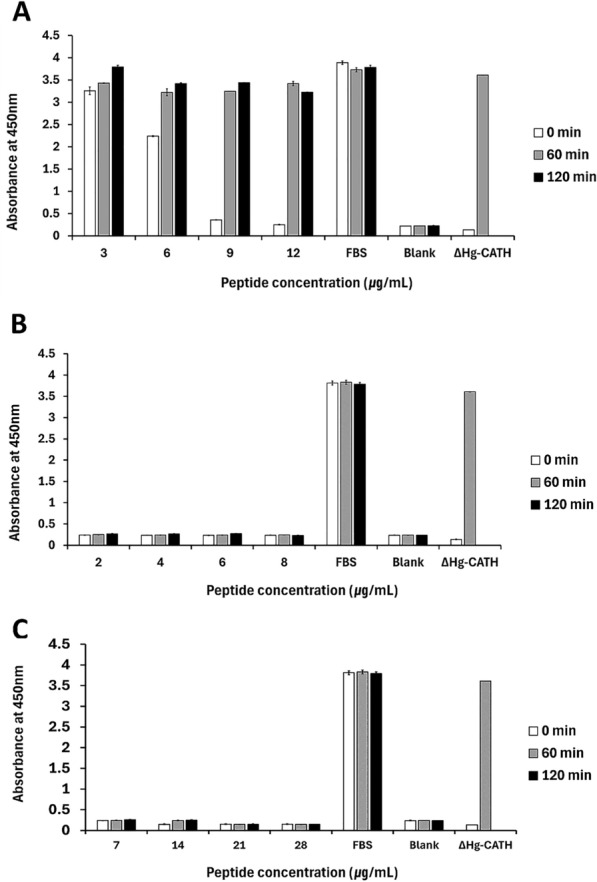
**Changes in the antibacterial activity of cc-CATH3, ML-CATH, and PD-CATH after incubation with naïve serum**. cc-CATH3, ML-CATH, and PD-CATH peptides were incubated in 50% (v/v) FBS each for 0, 60, and 120 min before applying to antimicrobial activity assay against *E. coli* (ATCC 25922). The antimicrobial activity was determined by measuring bacterial growth at OD 450 nm. The X and Y axes indicate peptide concentration and absorbance at 450 nm, respectively. Samples containing 50% FBS in the absence of the peptides indicate a control for bacterial growth without AMP treatments. The antimicrobial activity of cc-CATH3 (**A**), ML-CATH (**B**), and PD-CATH (**C**) after the treatment of FBS-incubated AMPs was shown.

Functional stability in the physiological condition is important for clinical therapeutics. Therefore, we tested variability in the antibacterial activity of cc-CATH3, PD-CATH, and ML-CATH against *E. coli* (ATCC 25922) in response to pH and salt condition changes. Interestingly, the bactericidal activity was marginally enhanced in more acidic conditions than in neutral conditions, revealing a decrease of MICs from 3 to 2 μg/mL (0.9 to 0.6 μM) and from 7 to 6 μg/mL (1.7 to 1.5 μM) for cc-CATH3 and PD-CATH, respectively. At the same time, ML-CATH was unaffected (2 μg/mL or 0.5 μM) by pH change and a high salt concentration (150 mM sodium chloride, Table 4). The bactericidal activity of cc-CATH3 was slightly decreased, revealing an increase of MICs from 3 to 5 μg/mL (0.9 to 1.6 μM) in a high salt condition. Additionally, AMPs were incubated with 50% naïve FBS for 0, 60, and 120 min, respectively. The changes in antimicrobial activities against *E. coli* (ATCC 25922) were determined by measuring the bacterial cell viability over incubation time. Based on a previous study [27], ΔHg-CATH was used as a reference for activity loss with serum incubation. For cc-CATH3, the antimicrobial activity of the peptides was significantly affected in concentrations lower than 3 × MIC (9 μg/mL or 2.7 μM) even in no prior incubation of the peptides with FBS (0 min) (Figure 2). However, the antibacterial activities of ML-CATH and PD-CATH remained unaffected under all the tested conditions. This outcome suggests that these two bat-derived AMPs are suitable for further exploration in relation to pharmaceutical applications.

**Table 4:** **Bactericidal activity of cc-CATH3, PD-CATH, and ML-CATH against** ***E. coli* under different pH and salinity**

Condition	Minimal inhibitory concentration (μg/mL, μM)		
	cc-CATH3	ML-CATH	PD-CATH
pH 5	2 (0.6)	2 (0.5)	6 (1.5)
pH 6	2 (0.6)	2 (0.5)	6 (1.5)
pH 7	3 (0.9)	2 (0.5)	7 (1.7)
150 mM NaCl	5 (1.6)	2 (0.5)	7 (1.7)
Control^a^	3 (0.9)	2 (0.5)	7 (1.7)

^a^Minimum inhibitory concentration (MICs) against *E. coli* (ATCC 25922) using Mueller–Hinton Broth (MHB) at pH 7.5.

### Low cytotoxic effects of cc-CATH3 and ML-CATH on mammalian cells

We then evaluated the viability of PK15 and HaCaT cells following incubation with 64 and 160 μg/mL of cc-CATH3, PD-CATH, and ML-CATH each for 24 h. The results were compared with those of melittin, which is highly cytotoxic to mammalian cells (Table 5). The cytotoxicity to PK15 and HaCaT at 64 μg/mL was minimal or unaffected, with cell viability ranging from 91.1 ± 1.8% to 100.6 ± 0.4% for either cc-CATH3 or ML-CATH. At the 160 μg/mL level, HaCaT viability was minimally affected with 90.1 ± 4.8% and 98.0 ± 1.5% for both cc-CATH3 and ML-CATH, respectively. However, the viability of PK15 cells was significantly reduced, with values of 32.7 ± 3.5% and 38.0 ± 2.2%, showing the dissimilarity in cytotoxic susceptibility of different cell types.

**Table 5:** **The influence of cc-CATH3, PD-CATH, and ML-CATH on the viability of mammalian cells under different concentrations**

Peptides	Concentration (μg/mL)	Cell viability ± SD (%)	
		PK15	HaCaT
cc-CATH3	64	91.1 ± 1.8	100.6 ± 0.4
	160	32.7 ± 3.5	90.1 ± 4.8
ML-CATH	64	96.2 ± 0.4	99.7 ± 0.9
	160	38.0 ± 2.2	98.0 ± 1.5
PD-CATH	64	43.6 ± 1.7	100.2 ± 0.2
	160	7.4 ± 0.2	11.4 ± 0.5
Melittin	64	7.7 ± 0.2	7.2 ± 0.1
Triton X-100 (1%)^a^		13.3 ± 0.1	13.0 ± 0.4

^a^A control for complete cell death.

The experiment was repeated three times.

For PD-CATH, the treatment of 64 μg/mL peptides showed survivability of 43.6 ± 1.7% and 100.22 ± 0.4% for PK15 and HaCaT, respectively. The cytotoxicity at 160 μg/mL was much higher than that of the other two AMPs, with cell viability of 7.4 ± 0.2% and 11.4 ± 0.5% for the corresponding cells. When cells were treated with 64 μg/mL melittin as a positive control, their survivability was ~ 7%. Considering the MICs of cc-CATH3, PD-CATH, and ML-CATH are < 10 μg/mL across most of the tested bacterial strains, cc-CATH3 and ML-CATH cytotoxicity can be minimal to mammalian cells.

Additionally, we analysed the haemolytic effect of cc-CATH3, PD-CATH, and ML-CATH against bovine erythrocytes at different concentrations ranging from 10 to 160 μg/mL (Table 6). Melittin caused hemolysis in > 90% of erythrocytes, even at the lowest level (10 μg/mL). In contrast, cc-CATH3 and PD-CATH exhibited a negligible level of haemolytic activity (2.3 ± 0.4% to 4.0 ± 1.0%) at the highest level (160 μg/mL). The haemolytic activity of ML-CATH ranged from 5.8 ± 0.4 to 11.8 ± 4.5%, which is greater than that of cc-CATH3 and PD-CATH.

**Table 6:** **Haemolytic activities of cc-CATH3, PD-CATH and ML-CATH against bovine erythrocytes**

Concentration (μg/mL)	cc-CATH3	ML-CATH	PD-CATH	Melittin^a^
10	0.0 ± 0.1	5.8 ± 0.4	0.4 ± 0.2	91.6 ± 1.0
20	0.0 ± 0.1	6.4 ± 0.8	0.7 ± 0.4	99.4 ± 0.9
40	0.4 ± 0.2	7.0 ± 0.8	2.9 ± 0.2	99.0 ± 0.3
80	0.8 ± 0.1	7.7 ± 0.2	3.5 ± 0.2	99.1 ± 0.5
160	2.3 ± 0.4	11.8 ± 4.5	4.0 ± 1.0	99.5 ± 0.3

The experiment was repeated three times.

^a^A control for complete cell lysis.

### Positive correlation of cationic charges to the potency of antimicrobial peptides

In this study, we compared the physicochemical characteristics of all evaluated AMPs to the antibacterial activity of the peptides against *E. coli* ATCC 25922 due to their strong activity (average of MICs = 10.21 μg/mL or 3.11 μM) in opposing the strain without exception (Table 2).

**Figure 3:**
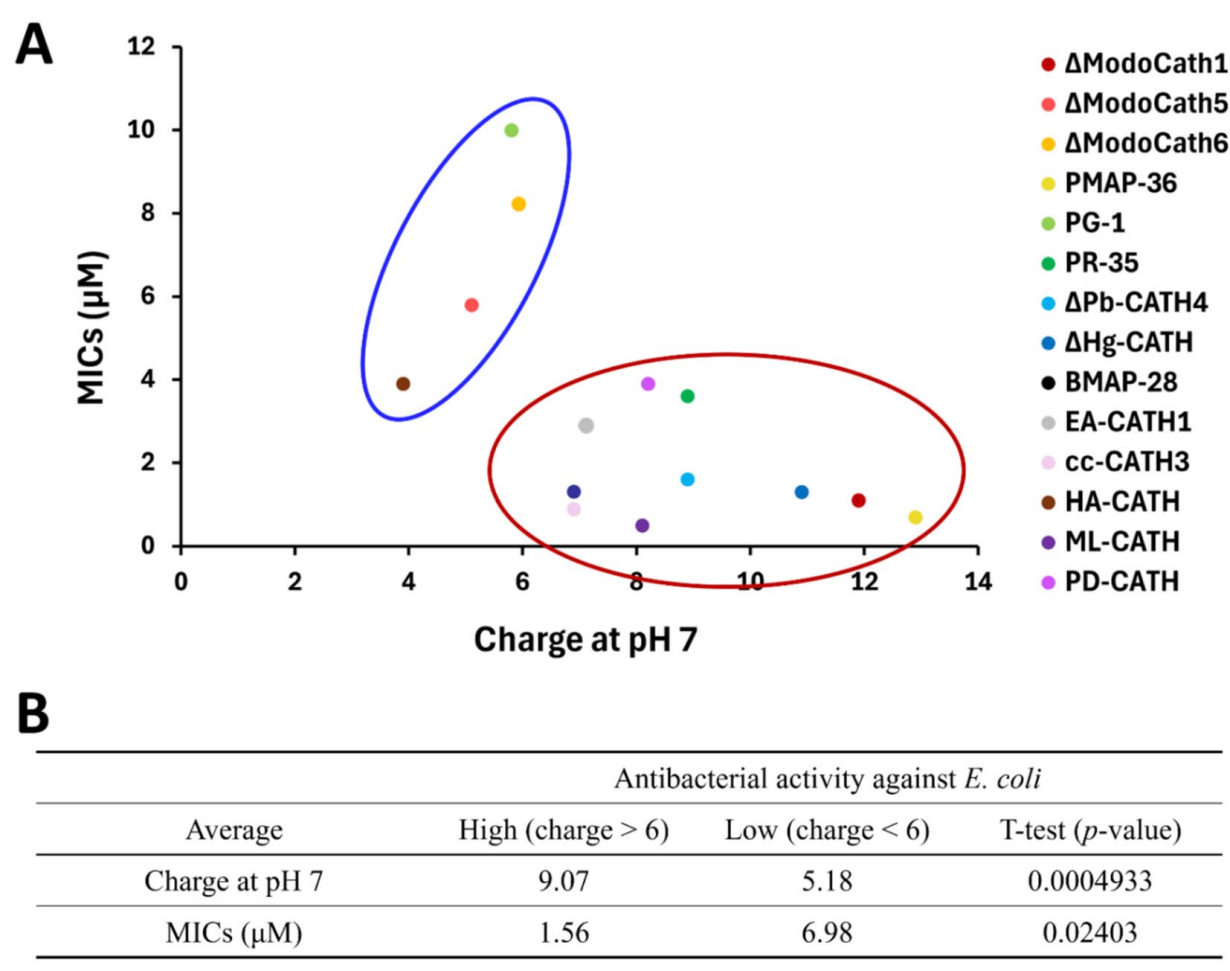
**Relationship between the strength of cationic charge and antibacterial activity among 14 cathelicidins**. **A** The minimum inhibition concentration (MIC) was differently distributed around + 6 charge and indicated by red and blue circles. **B** The average of charge and MICs in each group were calculated as shown. Welch’s *t* test was used for statistical analysis.

**Figure 4:**
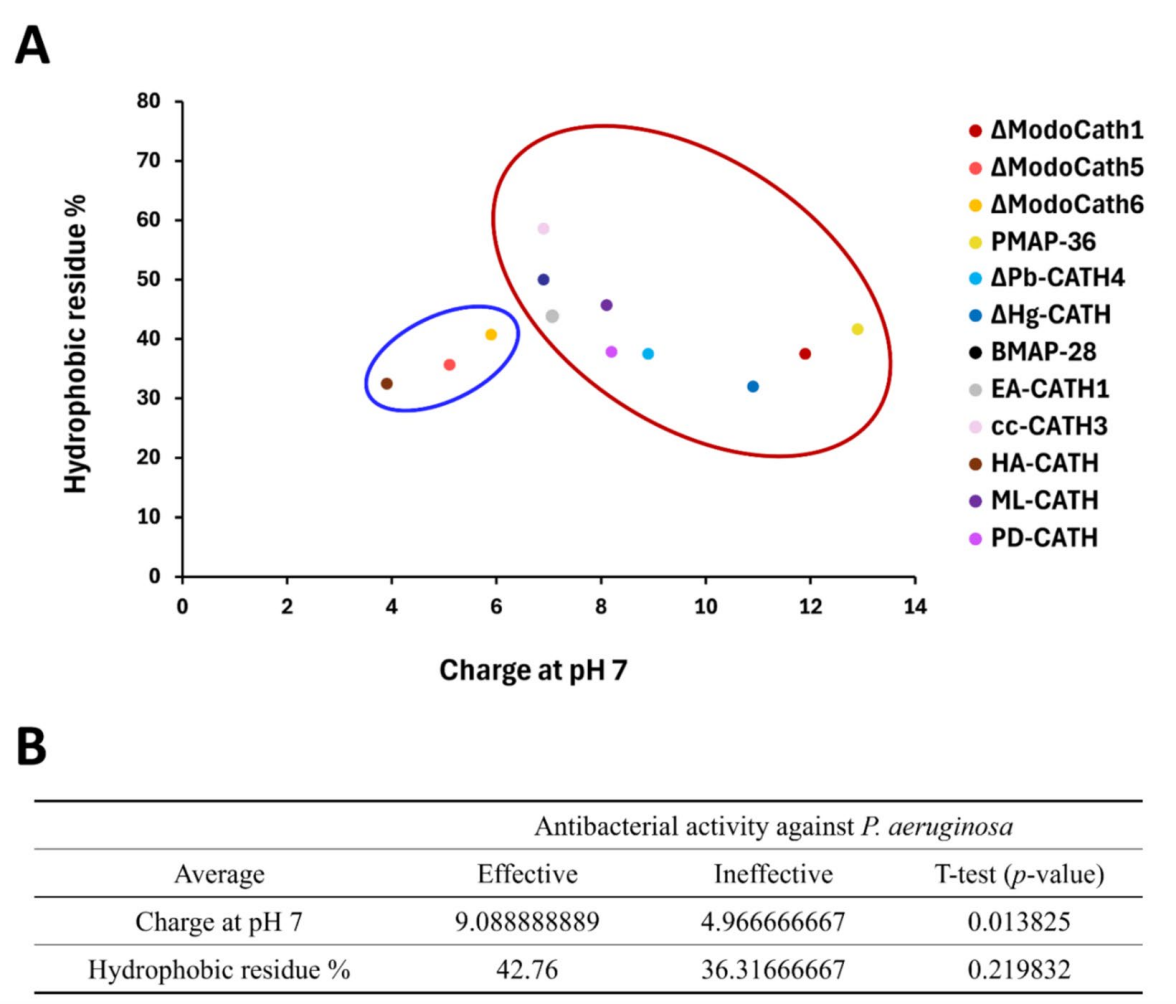
**Comparison of the strength of charge and hydrophobic residue proportion among helical cathelicidins**. **A** The antibacterial activity (MICs) of cathelicidins against *P. aeruginosa* (ATCC 27853) was divided into effective (< 45 μg/mL, red circle) and ineffective (> 40 μg/mL, blue circle) groups (Table 2 and Additional file 2). **B** The average charge and hydrophobic residue proportion for each group were calculated. as shown in the table at the bottom. Welch’s t-test was used for statistical analysis.

Our results suggest that peptides with higher positive charges (> + 6) had significantly lower MICs than those with lower charges (*p* value < 0.05, Figure 3). Further analysis on 12 helical cathelicidins in our panel also showed that cationic charge at pH 7 differs significantly between the potent and less potent peptide groups against *P. aeruginosa* (*p* value < 0.05) with the average values of 9.09 (effective group) and 4.97 (ineffective group), respectively (Figure 4 and Additional file 2). However, the other physicochemical properties, including the percentage of cationic and hydrophobic residues of the peptides, did not significantly correlate with the MICs (all ∣r∣ < 0.5) (Additional files 3A and B). The percentage of cationic and hydrophobic residues in the peptides was predominantly distributed between the 20–50% ranges. However, most peptides were longer than 20 amino acids (Additional file 3D). This finding is consistent with previous studies [50].

### Presence of Nonpolar_(n≥3)_-Polar_(n≥1)_-Nonpolar_(n≥3)_ motif in highly potent cathelicidins

**Figure 5:**
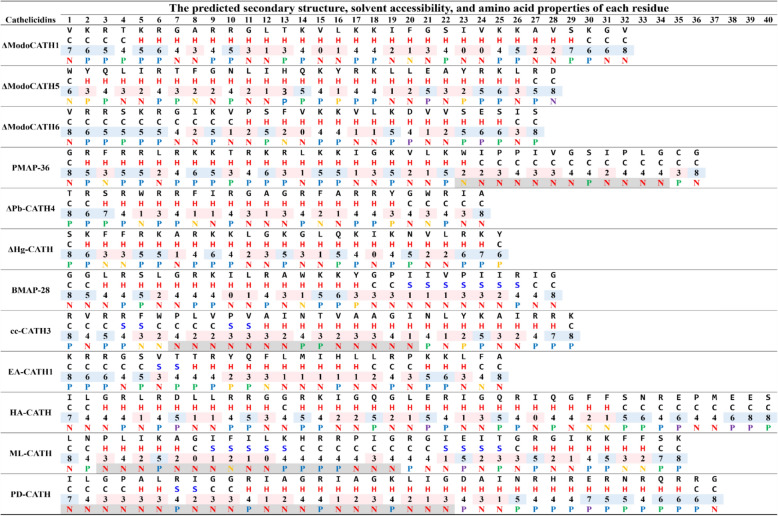
**Comparison of amino acid sequence and structural characteristics of helical cathelicidins**. Amino acid sequences are shown in the first row of each peptide. The predicted secondary structure (H: helix, S: strand, and C: coil) and solvent accessibility (values range from 0 (buried) to 8 (highly exposed residues)) for the individual residues are shown in the second and third rows of each peptide, respectively. The chemical properties corresponding to each amino acid are indicated in the fourth row of each peptide by different colors, as indicated in parentheses in the following description. “N” and “P” letters indicated nonpolar and polar, respectively. Nonpolar residues contain tryptophan (yellow), phenylalanine (yellow), and aliphatic amino acids (red), while polar residues include uncharged polar (green), positively (blue), and negatively (purple) charged residues and tyrosine (yellow). The unique motif, “N_(n≥3)_-P_(n≥1)_-N_(n≥3)_” was highlighted in grey.

The predicted secondary structures of the helical cathelicidins selected in this study showed high variability in the length and average SA of the helical, strand, and coil regions (Figure 5). In more detailed analysis, we observed the presence of a unique motif “N_(n≥3)_-P_(n≥1)_-N_(n≥3)_” (Nonpolar_(n≥3)_-Polar_(n≥1)_-Nonpolar_(n≥3)_) from PMAP-36, cc-CATH3, ML-CATH, and PD-CATH, which are AMPs with potent and broad-spectrum activities. The motif was observed in the helical or strand regions of cc-CATH3, ML-CATH, and PD-CATH, whereas in PMAP-36, it was observed in the C-terminal coil region. Additionally, ML-CATH and PD-CATH feature the motif multiple times (see Figure 5).

## Discussion

Bovine mastitis can be classified into subclinical and clinical forms according to the severity of the inflammation [51]. The degree of inflammation depends on the nature of causative pathogens, the age and breed of animals, their immunological health, and the lactation stage of cows [52]. Typically, antibiotic therapy is used to control infectious mastitis. However, the appearance of antimicrobial resistance in mastitis-associated pathogens such as methicillin-resistant *S. aureus* (MRSA), combined with the environment of dairy farms that act as a reservoir for antimicrobial-resistant pathogens, raises concerns about the excessive use of antibiotics [53–56]. Therefore, it is necessary to find therapeutic alternatives to antibiotic therapy for controlling the microorganisms that cause mastitis while minimising the risk of developing drug resistance.

This study underlines that AMPs could be used as possible alternatives to conventional antibiotics due to their broad-spectrum activities and, by their mechanisms, the rare probability of inducing antibiotic resistance [57]. We aimed to identify potent endogenous AMPs from animals that can fight bacterial species associated with contagious and environmental mastitis and to define their biochemical characteristics. Among the AMPs tested, ML-CATH was the most effective in controlling the diverse mastitis-associated bacterial strains with minimal cytotoxicity to mammalian cells. During the study, we observed a unique motif “N_(n≥3)_-P_(n≥1)_-N_(n≥3)_” from the sequences of PMAP-36, cc-CATH3, ML-CATH, and PD-CATH with broader and stronger antimicrobial activities than the other AMPs. To our knowledge, this is the first report comparing the bactericidal activities against numerous bovine-mastitis causative bacterial species, including field isolates.

Founded on the previously established concept that cationic and hydrophobic residues of AMPs are aligned at opposite sites on their helical wheel projection, amino acid substitutions for endogenous or synthetic AMPs have inevitably been conducted accordingly. These substitutions aimed to improve the hydrophobic moment, amphipathicity, and helicity to increase antimicrobial and potentially reduce cytotoxic effects [19–21]. However, recent studies have indicated that these factors have a low correlation with antimicrobial activity and spectrum [21]. Furthermore, cc-CATH, PD-CATH, and ML-CATH, with the broadest activity spectrum and highest potency in our AMP panel, consistently presented relatively low hydrophobic moment (0.271, 0.180, and 0.372, respectively) and a less clear amphipathicity in their helical wheel projection (Table 2 and Figure 1). Conversely, the charge of AMPs appears to be a major determinant for killing Gram-negative bacteria such as *E. coli* and *P. aeruginosa.*

Previous research has also reported that the charge of AMPs acts as an important factor contributing to salt insensitivity [38]. Thus, it has been understood that highly positively charged AMPs could compete with cations and interact with the outer membrane more effectively [58]. Despite this previous reporting, the factors largely influencing antibacterial activities against Gram-positive bacteria remained unclear in our analysis, which might be attributed to the high complexity and diversity of their cell wall composition and structure compared to those of Gram-negative bacteria [59].

In this study, the modified PMAP-36 analogs, excluding the N_(n≥3)_-P_(n≥1)_-N_(n≥3)_ motif in the C-terminal region, showed weaker antibacterial activity and hemolysis than the parental peptide [60]. Additionally, ΔPD-CATH and ΔML-CATH, truncated forms of PD-CATH and ML-CATH, dramatically lost antimicrobial activity and cytotoxicity [32, 60]. This motif was also identified from other Aves, Reptilia, Amphibia, and Mammalia cathelicidins in public databases (Additional file 4). Therefore, further experimental validation is required to corroborate the functional importance of the N_(n≥3)_-P_(n≥1)_-N_(n≥3)_ motif.

Unlike ML-CATH, cc-CATH and PD-CATH shared structural properties with a large helix from the middle to C-terminal regions (Figure 5 and Additional file 5). As suggested previously [20], many polar residues in their C-terminal helix might be associated with higher cytotoxicity to mammalian cells than ML-CATH (Tables 5 and 6). It has been previously presented that the cytotoxicity of AMPs in mammalian cells varies depending on cell types, even within a species [23]. We observed similar results from PK15 and HaCaT (Table 5). Interestingly, the secondary structure of ML-CATH was symmetrical as “coil-helix-strand-coil-strand-helix-coil” (Figure 5). This structure may provide greater structural flexibility than other AMPs by offering a continuous large helix such as ΔModoCATH1, ΔModoCATH5, ΔPb-CATH4, and ΔHg-CATH to interact with bacterial membranes or cell walls.

Various AMPs are expressed in mammary epithelial cells and are upregulated in naturally occurring and experimentally induced mastitis [42, 46, 61, 62]. Among bovine cathelicidins, Bac5, indolicidin, and BMAP-28 expression was detected in somatic cells extracted from milk samples taken from cows with high SCCs. However, the expression patterns of the cathelicidin genes were significantly variable between animals [45]. For example, the expression levels of cathelicidins could depend on the duration of lactation, the presence of an infection, and the types of causative bacteria. BMAP-27 and BMAP-28 showed effective antibacterial activity against *E. coli* in standard bacterial growth media, blood serum, and whey [46]. Among the bovine cathelicidins, BMAP-28 mRNA was detected in bovine alveolar, ductal, gland cistern, and teat canal tissues from the mammary gland of healthy and experimentally infected animals with *S. aureus* [45]. It is proposed that the expression of multiple cathelicidins could, therefore, provide a synergistic effect [46].

Similar to the theory of controlling inflammatory processes using AMPs, bacteriocins can also be suggested as an approach against mastitis pathogens. Nisin, a commercially available bacteriocin produced by *Lactococcus lactis*, exhibited its inhibitory effect on several Gram-positive pathogens associated with mastitis (Table 2). This finding is consistent with previous research [40]. Another study indicated that, because of nisin’s therapeutic applicability, the activity of a formulation with the peptide was comparable with chemical germicides but without skin irritation. Such an outcome can be determined by measuring the germicidal activity against mastitis pathogens on the teat skin of live cows [63].

Due to the anti-staphylococcal activity of lysostaphin, a bacteriocin produced by *Staphylococcus simulans*, we evaluated its antimicrobial effect against mastitis-associated bacteria and staphylococci in the field isolates. Based on the bacterial profiles in field isolates, staphylococci are the main causative pathogens of bovine mastitis (Table 3). Moreover, as shown in Table 2 and Additional file 6, lysostaphin showed highly specific activities at the nanomolar range against staphylococci, which could suggest its therapeutic application. As the antibacterial activity of lysostaphin has a narrow spectrum, it may be more beneficial to utilise a combination of lysostaphin and other AMPs in treatment.

A study on the identification of mastitis pathogens in Korea classified staphylococci (52.9%) and Gram-negative bacilli (other than *Escherichia coli*, 19.5%) as major pathogens. The study also classified *Streptococcus uberis* (5.3%), *Enterococcus* spp. (4.8%), *E. coli* (4.5%), *Aerococcus* spp. (2.6%), and *Corynebacterium* spp. (1.2%) as minor pathogens [64]. The classifications of field isolates in our study were consistent with the abovementioned classifications, except for *Aeromonas hydrophila* (Additional file 1). According to CLSI breakpoints, most of the field isolates in this study displayed resistance to ampicillin based on their MICs (CLSI breakpoints ≥ 0.25 μg/mL) when compared to non-resistant strains of staphylococci (Table 3). This finding is consistent with previous studies [65]. Staphylococci, *Corynebacterium simulans*, and *A. hydrophila* were found to be susceptible to gentamicin and chloramphenicol, with MICs ranging between susceptible and intermediate levels. At the same time, *Streptococcus uberis* resisted all antibiotics (Table 3).

In contrast, cc-CATH3, ML-CATH, and PD-CATH showed highly potent bactericidal activity at a nanomolar range (MICs: 0.8–5 μg/mL or 0.2–1.2 μM) against all field isolates except *A. hydrophila*. Interestingly, Bat AMPs, such as PD-CATH and ML-CATH, showed a relatively weak activity (MIC: 160 μg/mL) against the strain, which is a Gram-negative species found as a pathogen in reptiles, amphibians, fish, cows, and humans [66]. It has previously been reported that *A. hydrophila* showed resistance to diverse antibiotics such as erythromycin, amoxicillin, oxytetracycline, gentamicin, and kanamycin [67]. Furthermore, in a previous study analysing the biological activities of AMPs from the skin of three frog species, bactericidal activity against *A. hydrophila* was not observed [66]. These findings suggest that endogenous AMPs of animals are poorly active against *A. hydrophila.*

However, despite AMPs being a promising antimicrobial agent that can be used as an alternative to chemical antibiotics, more efforts are necessary to overcome several inherent limitations of the molecules, including high production costs, sensitivity to proteolytic degradation, and mammalian cell cytotoxicity. In this study, we successfully produced recombinant PG1 using previously reported production methods [22, 23, 68]. In doing so, we demonstrated that large-scale production of recombinant AMPs could be achieved. Moreover, few studies have investigated the in vivo efficacy of endogenous AMPs. The clinical effects of bovine lactoferrin (bLf) on staphylococcal mastitis were previously only evaluated by the intramammary infusion method, which showed that the number of staphylococci in the mammary gland was decreased in the bLf-infused group compared to that of antibiotic therapy [69]. Therefore, historically, a gene therapy strategy to treat bovine mastitis was developed using a mammary-specific vector expressing human lysozyme (hLYZ) [70].

In this study, we presented the antibacterial activities of 16 AMPs against diverse bacterial species. Our objective was to screen promising candidates for mastitis therapeutics as an alternative to conventional antibiotics. Consequently, we demonstrated that ML-CATH could be exploited as an effective therapeutic agent in antimicrobial activity and spectrum, cytotoxicity, and physiological stability. This finding indicates that the combinatorial effects of multiple AMPs against bacterial species that show weak or no bactericidal response from treating an individual AMP may result in improved responses through synergistic effects.

Our conclusions from comparing the biochemical features of AMPs with their biological activities further illuminate relationships between their physicochemical structure and biological activity. The information on the antimicrobial properties of AMPs in this study provides a valuable contribution to developing AMP-based therapeutic agents to control various contagious bacterial diseases, including bovine mastitis.

## Supplementary Information

**Additional file 1. Identification of the field isolates from milk with high SCCs using microbiological analysis and 16S rRNA gene sequencing**. The bacterial strains were isolated from culturing milk samples of dairy cows with high SCC (≥ 500,000 cells/mL) and analysed microbiological properties assessing growth, colony morphologies, and haemolytic characteristics on the blood agar plate. The isolated bacterial colonies were used to sequence the 16S rRNA gene. NCBI blast analysis against rRNA/ITS databases using the sequencing results matched to 16S rRNA sequences of known bacterial species with coverage of > 96% and identity of > 97%. The clone IDs were named “the individual number-teat position (left or right/front or rear).

**Additional file 2. Classification of analysed helical cathelicidins into two different groups according to their antimicrobial activity**. The peptides were classified into two distinct groups, “Effective” (MICs < 45 μg/mL) and “Ineffective” (MICs > 40 μg/mL) for correlation analysis. To compare the physicochemical characteristics of all helical cathelicidins evaluated in the current study to the antibacterial activity of these peptides against each bacterial strain.

**Additional file 3. Relationship between biochemical characteristics and antibacterial activity of cathelicidins**. The percentages of (A) cationic residues, (B) hydrophobic residues, and (C) the peptide length (X-axis) were compared to MIC values (Y-axis) against *E. coli* (ATCC 25922). Each peptide was presented in different colours.

**Additional file 4. Comparison of amino acid sequence and structural characteristics of 48 selected cathelicidins available in DBAASP**. (A) Amino acid sequences are shown in the first row of each peptide. The chemical properties corresponding to each amino acid are indicated in the second row of each peptide by different colours, as indicated in parentheses in the following description. “N” and “P” letters indicated nonpolar and polar, respectively. Nonpolar residues contain tryptophan (yellow), phenylalanine (yellow) and aliphatic amino acids (red), while polar residues include uncharged polar (green), positively (blue) and negatively (purple) charged residues and tyrosine (yellow). The unique motif, “N_(n≥3)_-P_(n≥1)_-N_(n≥3)_” was highlighted in grey. ID, accession ID for DBAASP. (B) Analysed cathelicidins were classified into four taxonomic groups: Aves, Reptilia, Amphibia and Mammalia. The number of the peptides containing the motif was presented in the column of “N_(n≥3)_-P_(n≥1)_-N_(n≥3)_ Motif”, and their ratio to the total number of peptides in each group shown the list was calculated.

**Additional file 5. Tertiary structure modelling by I-TASSER**. The tertiary structures of (A) cc-CATH3, (B) PD-CATH and (C) ML-CATH were predicted and then visualised by PyMOL as the strand structure of ML-CATH was not presented by the visualisation of PyMOL. The direction of N- to C-termini in their structures was indicated by blue to red.

**Additional file 6. Antibacterial activity of lysostaphin against staphylococci from field isolation**. The anti-staphylococcal activity of lysostaphin was evaluated against the mastitis-associated staphylococci in the field isolates, considering that staphylococci are the main causative pathogens of bovine mastitis based on the bacterial profiles in field isolates.

## Data Availability

The datasets supporting the conclusions of this article are included within the article and its additional file.
