# Supplementary material for: Bactericidal activities and biochemical features of 16 antimicrobial peptides against bovine-mastitis causative pathogens
Source: Vet Res. 2024 Nov 14;55:150. doi: 10.1186/s13567-024-01402-x (PMC11566078; doi:10.1186/s13567-024-01402-x)
Supplement: Supplementary file 1 — Additional file 1. Identification of the field isolates from milk with high SCCs using microbiological analysis and 16S rRNA gene sequencing. The bacterial strains were isolated from culturing milk samples of dairy cows with high SCC (≥ 500,000 cells/mL) and analysed microbiological properties assessing growth, colony morphologies, and haemolytic characteristics on the blood agar plate. The isolated bacterial colonies were used to sequence the 16S rRNA gene. NCBI blast analysis against rRNA/ITS databases using the sequencing results matched to 16S rRNA sequences of known bacterial species with coverage of > 96% and identity of > 97%. The clone IDs were named “the individual number-teat position (left or right/front or rear). [file 13567_2024_1402_MOESM1_ESM.docx]

**Additional file 1. Identification of the field isolates from milk with high SCCs using microbiological analysis and 16S rRNA gene sequencing.**

|  |  | Colony | |  | Plate | |  | Sequence identity | |  | Blast matches | | |
| --- | --- | --- | --- | --- | --- | --- | --- | --- | --- | --- | --- | --- | --- |
| Clone ID^a^ | Hemolysis | Color | Size |  | MCA^b^ | MHA^c^ |  | Query cover | Identity |  | Description | Accession |  |
| 123LF-1, 132RR-1, 161LR-1 | β | Yellow | Big |  | X | O |  | 98% | 99.73% |  | *Staphylococcus chromogenes* | NR_036901.1 |  |
| 132LF-1 | β | Milk-white | Big |  | O | O |  | 99% | 99.87% |  | *Aeromonas hydrophila* | NR_074841.1 |  |
| 134RF-1 | β | Milk-white | Big |  | X | O |  | 98% | 99.80% |  | *Staphylococcus haemolyticus* | NR_113345.1 |  |
| 123RF-3, 211LR-2 | α | Green | Middle |  | X | O |  | 99% | 99.80% |  | *Aerococcus viridans* | NR_104708.1 |  |
| 196RF-3 | α | Green | Small |  | X | O |  | 99% | 98.84% |  | *Ruoffia tabacinasalis* | NR_026482.1 |  |
| 198RR-1 | None | Milk-white | Middle |  | X | O |  | 99% | 100.00% |  | *Staphylococcus xylosus* | NR_036907.1 |  |
| 178LR-3 | None | Gray | Middle |  | X | O |  | 99% | 99.93% |  | *Staphylococcus epidermidis* | NR_113957.1 |  |
| 152RF-2, 152LF-1 | None | White | Small |  | X | O |  | 98% | 98.96% |  | *Corynebacterium amycolatum* | NR_119171.1 |  |
| 178RR-3 | None | Yellow | Small |  | X | O |  | 99% | 97.40% |  | *Corynebacterium simulans* | NR_025309.1 |  |
| 211LR-1 | None | Green | Middle |  | X | O |  | 99% | 99.52% |  | *Glutamicibacter nicotianae* | NR_026190.1 |  |
| 218RF-2 | None | Gray | Small |  | X | O |  | 99% | 99.80% |  | *Streptococcus uberis* | NR_040820.1 |  |
| 123LF-2 | α | Brown | Small |  | X | O |  | 97% | 99.44% |  | *Chryseobacterium aquaticum* | NR_042642.1 |  |
| 123LF-4 | α | Green | Small |  | X | O |  | 96% | 99.51% |  | *Jeotgalibaca porci* | NR_156898.1 |  |

^a^ It was named as “the individual number-teat position (left or right/front or rear)”

^b^ MacConkey agar

^c^ Mueller-Hinton (MH) agar
