# Supplementary material for: Bactericidal activities and biochemical features of 16 antimicrobial peptides against bovine-mastitis causative pathogens
Source: Vet Res. 2024 Nov 14;55:150. doi: 10.1186/s13567-024-01402-x (PMC11566078; doi:10.1186/s13567-024-01402-x)
Supplement: Supplementary file 2 — Additional file 2. Classification of analysed helical cathelicidins into two different groups according to their antimicrobial activity. The peptides were classified into two distinct groups, “Effective” (MICs < 45 μg/mL) and “Ineffective” (MICs > 40 μg/mL) for correlation analysis. To compare the physicochemical characteristics of all helical cathelicidins evaluated in the current study to the antibacterial activity of these peptides against each bacterial strain. [file 13567_2024_1402_MOESM2_ESM.docx]

**Additional file 2. Classification of analyzed helical cathelicidins into two different groups according to their antimicrobial activity.**

|  | **Helical cathelicidins (*n*=12)** | |
| --- | --- | --- |
| **Bacteria** | **Effective group (MICs < 45 μg/mL)** | **Ineffective group (MICs > 40 μg/mL)** |
| *P. aeruginosa* | ΔModoCath1, PMAP-36, ΔPb-CATH4, ΔHg-CATH, BMAP-28, EA-CATH1, cc-CATH3, ML-CATH, PD-CATH | ΔModoCath5, ΔModoCath6, HA-CATH |
| *S. aureus* | ΔModoCath1, ΔModoCath5, PMAP-36, ΔPb-CATH4, BMAP-28, EA-CATH1, cc-CATH3, HA-CATH, ML-CATH, PD-CATH | ΔModoCath6, ΔHg-CATH |
| *B. cereus* | ΔModoCath5, PMAP-36, BMAP-28, cc-CATH3, HA-CATH, ML-CATH, PD-CATH | ΔModoCath1, ΔModoCath6, ΔPb-CATH4, ΔHg-CATH, EA-CATH1 |
| *E. faecalis* | ΔModoCath1, ΔModoCath5, BMAP-28, EA-CATH1, cc-CATH3, ML-CATH, PD-CATH | ΔModoCath6, PMAP-36, ΔPb-CATH4, ΔHg-CATH, HA-CATH |
| *S. agalactiae* | cc-CATH3, ML-CATH, PD-CATH | ΔModoCath1, ΔModoCath5, ΔModoCath6, PMAP-36, ΔPb-CATH4, ΔHg-CATH, BMAP-28, EA-CATH1, HA-CATH |
| *S. dysgalactiae* | ΔHg-CATH, cc-CATH3, ML-CATH, PD-CATH | ΔModoCath1, ΔModoCath5, ΔModoCath6, PMAP-36, ΔPb-CATH4, BMAP-28, EA-CATH1, HA-CATH |
| *S. eui* | ΔModoCath1, ΔPb-CATH4, ΔHg-CATH, cc-CATH3, ML-CATH, PD-CATH | ΔModoCath5, ΔModoCath6, PMAP-36, BMAP-28, EA-CATH1, HA-CATH |
