# Supplementary figures and images for: Bactericidal activities and biochemical features of 16 antimicrobial peptides against bovine-mastitis causative pathogens
Source: Vet Res. 2024 Nov 14;55:150. doi: 10.1186/s13567-024-01402-x (PMC11566078; doi:10.1186/s13567-024-01402-x)

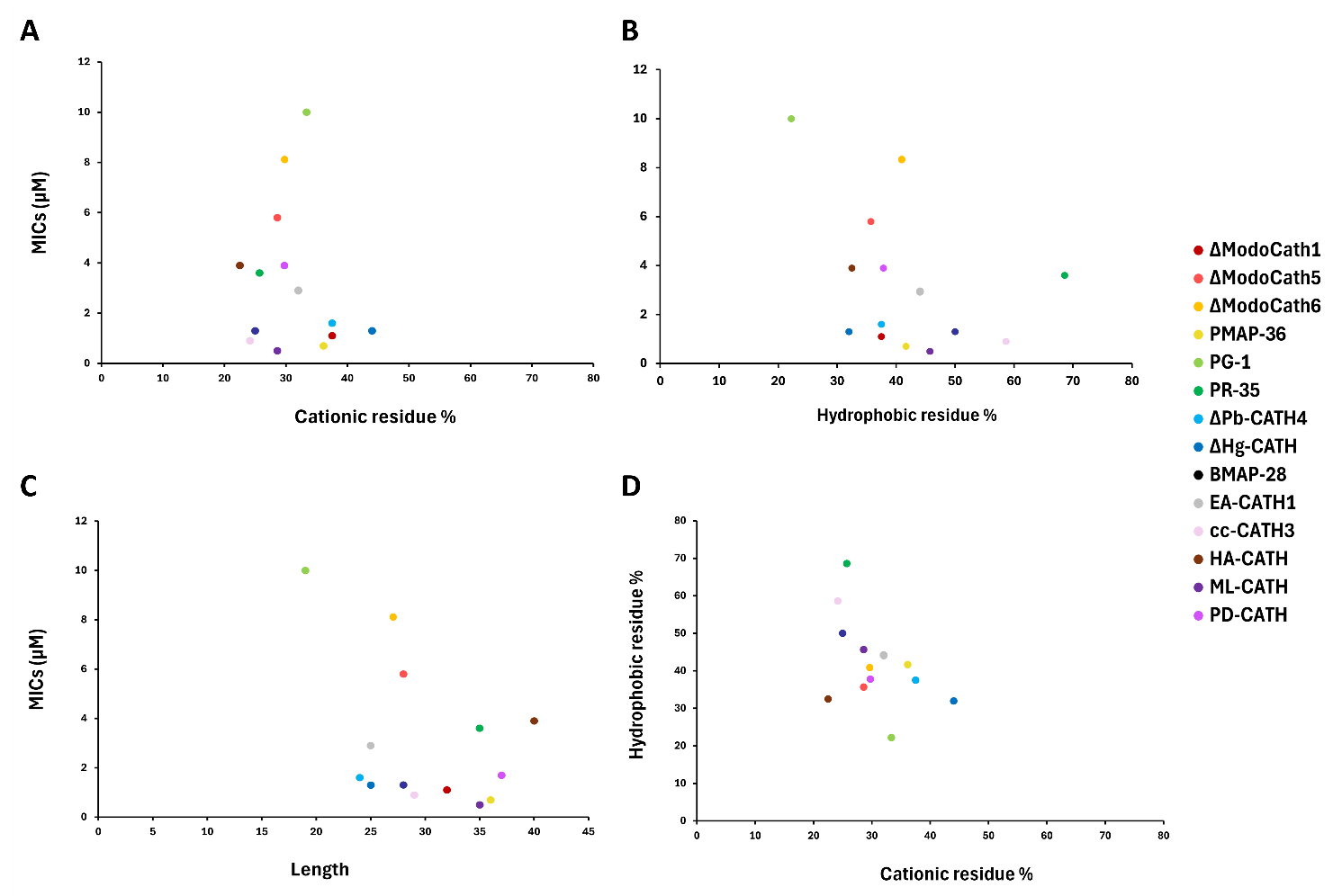

Supplement: Supplementary file 3 — Additional file 3. Relationship between biochemical characteristics and antibacterial activity of cathelicidins. The percentages of (A) cationic residues, (B) hydrophobic residues, and (C) the peptide length (X-axis) were compared to MIC values (Y-axis) against E. coli (ATCC 25922). Each peptide was presented in different colours. [file 13567_2024_1402_MOESM3_ESM.docx]

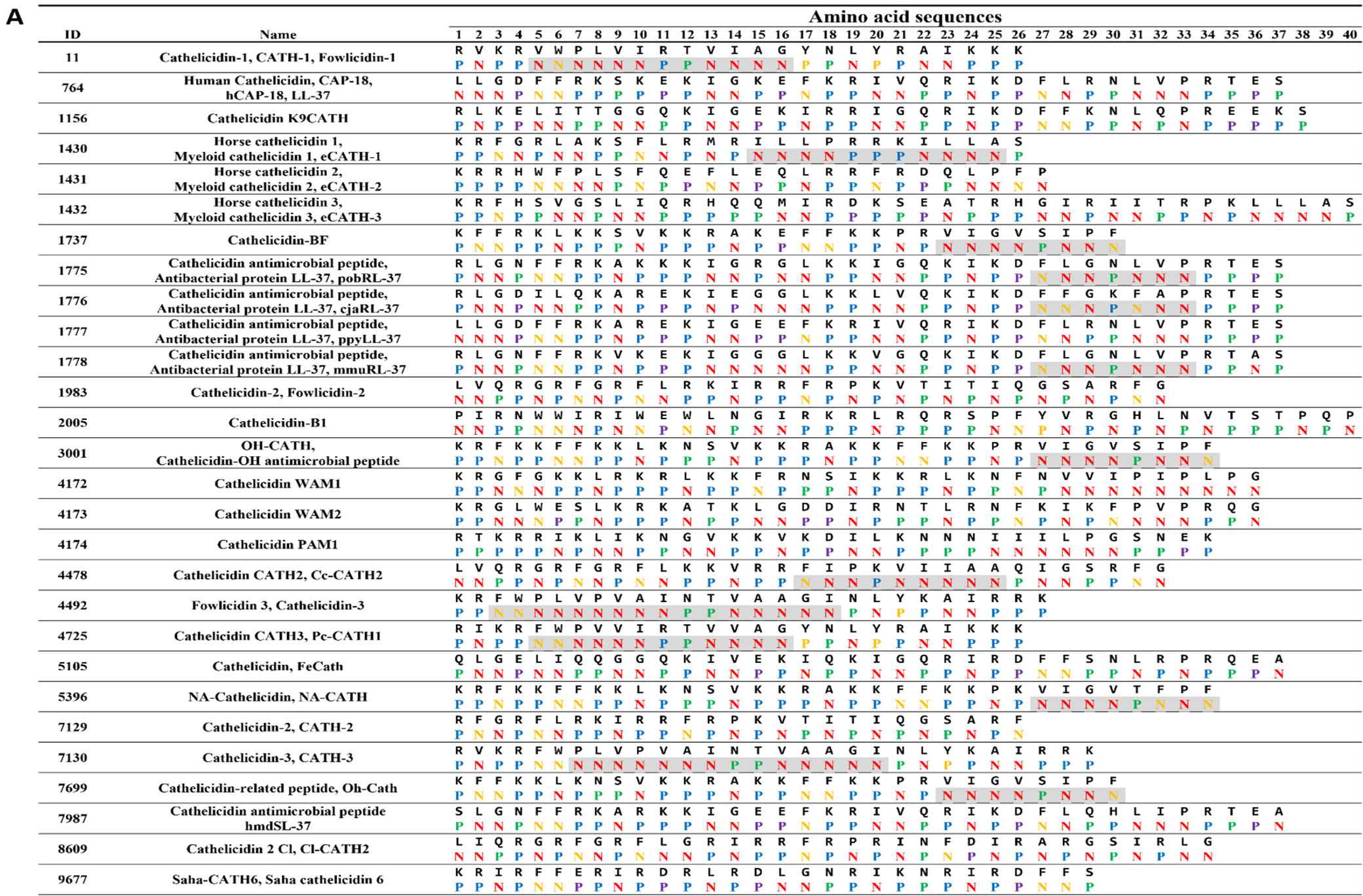


(Continued)
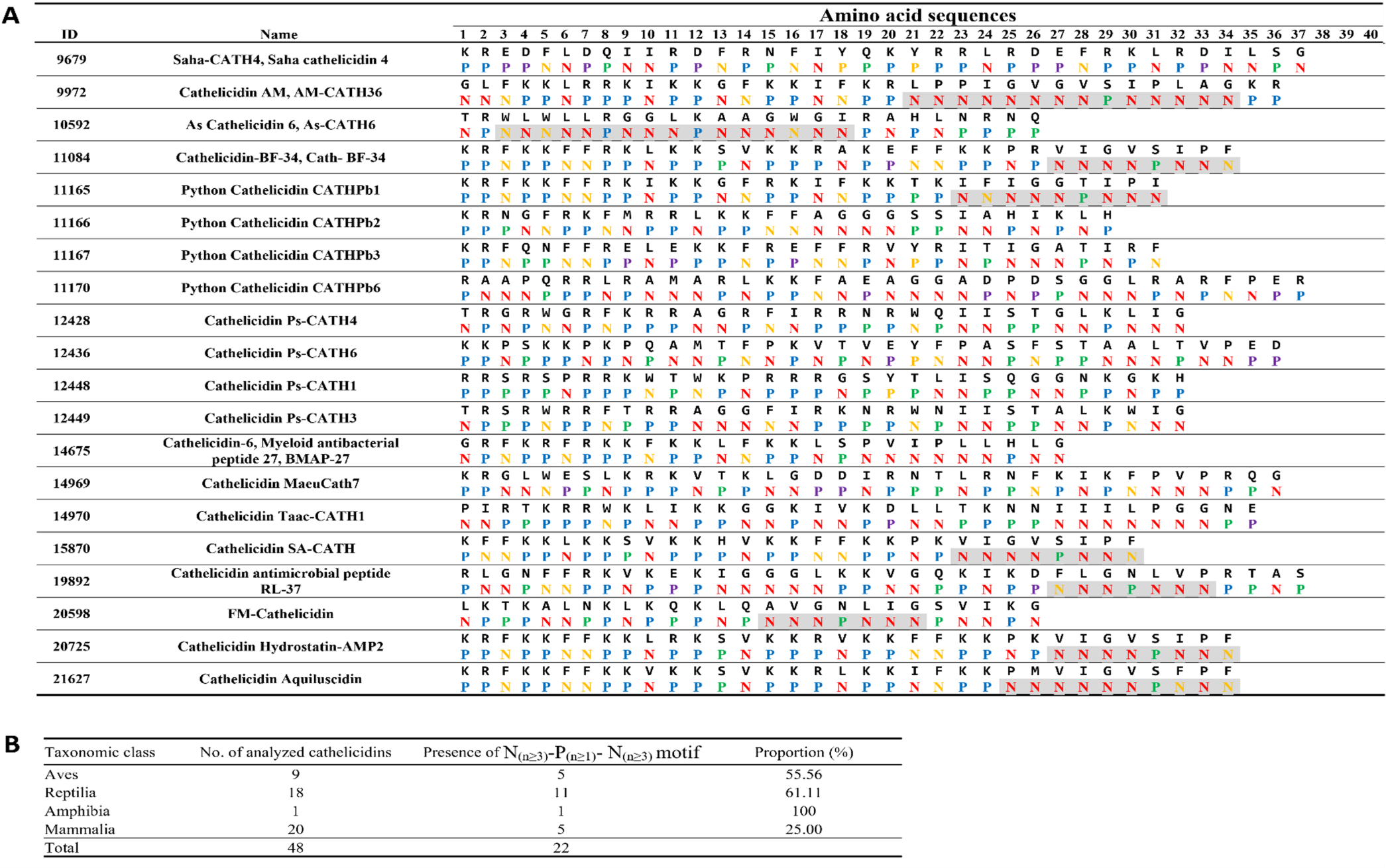

Supplement: Supplementary file 4 — Additional file 4. Comparison of amino acid sequence and structural characteristics of 48 selected cathelicidins available in DBAASP. (A) Amino acid sequences are shown in the first row of each peptide. The chemical properties corresponding to each amino acid are indicated in the second row of each peptide by different colours, as indicated in parentheses in the following description. “N” and “P” letters indicated nonpolar and polar, respectively. Nonpolar residues contain tryptophan (yellow), phenylalanine (yellow) and aliphatic amino acids (red), while polar residues include uncharged polar (green), positively (blue) and negatively (purple) charged residues and tyrosine (yellow). The unique motif, “N(n≥3)-P(n≥1)-N(n≥3)” was highlighted in grey. ID, accession ID for DBAASP. (B) Analysed cathelicidins were classified into four taxonomic groups: Aves, Reptilia, Amphibia and Mammalia. The number of the peptides containing the motif was presented in the column of “N(n≥3)-P(n≥1)-N(n≥3) Motif”, and their ratio to the total number of peptides in each group shown the list was calculated. [file 13567_2024_1402_MOESM4_ESM.docx]

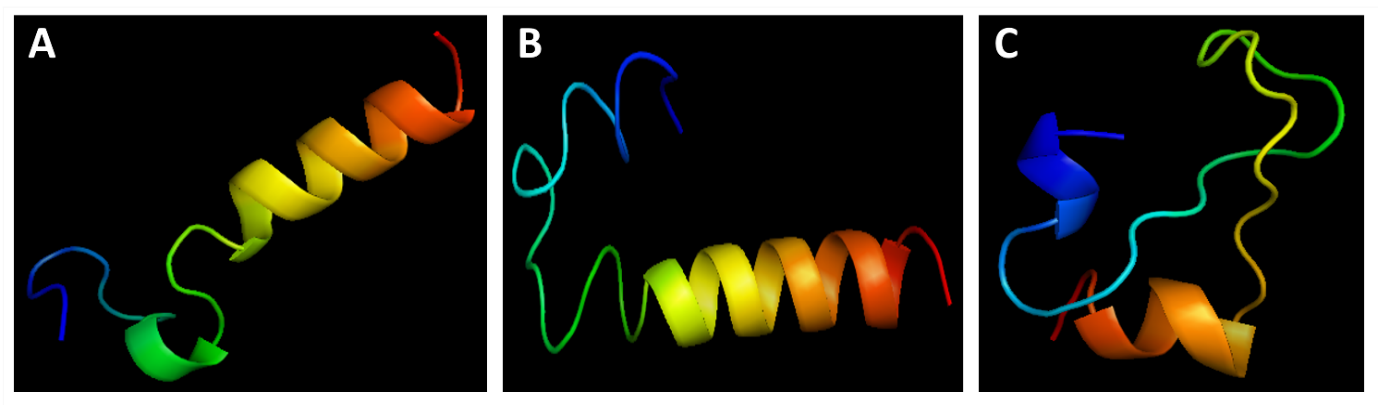

Supplement: Supplementary file 5 — Additional file 5. Tertiary structure modelling by I-TASSER. The tertiary structures of (A) cc-CATH3, (B) PD-CATH and (C) ML-CATH were predicted and then visualised by PyMOL as the strand structure of ML-CATH was not presented by the visualisation of PyMOL. The direction of N- to C-termini in their structures was indicated by blue to red. [file 13567_2024_1402_MOESM5_ESM.docx]
