# Supplementary material for: Bactericidal activities and biochemical features of 16 antimicrobial peptides against bovine-mastitis causative pathogens
Source: Vet Res. 2024 Nov 14;55:150. doi: 10.1186/s13567-024-01402-x (PMC11566078; doi:10.1186/s13567-024-01402-x)
Supplement: Supplementary file 6 — Additional file 6. Antibacterial activity of lysostaphin against staphylococci from field isolation. The anti-staphylococcal activity of lysostaphin was evaluated against the mastitis-associated staphylococci in the field isolates, considering that staphylococci are the main causative pathogens of bovine mastitis based on the bacterial profiles in field isolates. [file 13567_2024_1402_MOESM6_ESM.docx]

**Additional file 6. Antibacterial activity of lysostaphin against staphylococci from field isolation.**

|  |  | Minimal inhibitory concentration (μg/mL, nM) | | | |
| --- | --- | --- | --- | --- | --- |
|  | Strains | Lysostaphin | Chloramphenicol^a^ | Ampicilin^a^ | Gentamicin^a^ |
| Field isolates | *Staphylococcus chromogenes* | 0.25 (9.3) | 18 (55.8) | 5 (14.3) | 2 (4.2) |
|  | *Staphylococcus haemolyticus* | 1 (37.3) | 10 (31.0) | 10 (28.6) | 10 (20.9) |
|  | *Staphylococcus xylosus* | 0.5 (18.7) | 8 (24.8) | 5 (14.3) | 1 (2.1) |
|  | *Staphylococcus epidermidis* | 1 (37.3) | 5 (15.5) | 5 (14.3) | 4 (8.4) |

^a^ Antibiotics for control.
